# Supplementary material for: Women’s lived experiences of sex hormones and life-related to bariatric surgery: an interpretative qualitative study
Source: BMJ Open. 2023 Jun 23;13(6):e072085. doi: 10.1136/bmjopen-2023-072085 (PMC10314659; doi:10.1136/bmjopen-2023-072085)
Supplement: Author's manuscript [file bmjopen-2023-072085.draft_revisions.pdf]

BMJ Open

BMJ Open is committed to open peer review. As part of this commitment we make the peer review history of every article we publish publicly available.

When an article is published we post the peer reviewers' comments and the authors' responses online. We also post the versions of the paper that were used during peer review. These are the versions that the peer review comments apply to.

The versions of the paper that follow are the versions that were submitted during the peer review process. They are not the versions of record or the final published versions. They should not be cited or distributed as the published version of this manuscript.

BMJ Open is an open access journal and the full, final, typeset and author-corrected version of record of the manuscript is available on our site with no access controls, subscription charges or pay-per-view fees (http://bmjopen.bmj.com).

If you have any questions on BMJ Open's open peer review process please email info.bmjopen@bmj.com

BMJ Open

Fertile women's lived experiences of life before and after bariatric surgery: An interpretative qualitative study

Journal:	*BMJ Open*
Manuscript ID	bmjopen-2023-072085
Article Type:	Original research
Date Submitted by the Author:	20-Jan-2023

Complete List of Authors:	Paul, Rebecca; Linköping University, Department of Surgery Department of Biomedical and Clinical Sciences; Center for Clinical Research Dalarna, Department of Surgery, Falun County Hospital<br>Andersson, Ellen; Linköping University, Department of Surgery Department of Biomedical and Clinical Sciences<br>Olbers, Torsten ; Linköping University<br>Frisk, Jessica; Linköping University, Department of Surgery Department of Biomedical and Clinical Sciences<br>Berterö, Carina; Linköping University, Institution of Medical and health Sciences

Keywords:	Sex steroids & HRT < DIABETES & ENDOCRINOLOGY, QUALITATIVE RESEARCH, Quality of Life, Adult surgery < SURGERY

SCHOLARONE™
Manuscripts

I, the Submitting Author has the right to grant and does grant on behalf of all authors of the Work (as defined in the below author licence), an exclusive licence and/or a non-exclusive licence for contributions from authors who are: i) UK Crown employees; ii) where BMJ has agreed a CC-BY licence shall apply, and/or iii) in accordance with the terms applicable for US Federal Government officers or employees acting as part of their official duties; on a worldwide, perpetual, irrevocable, royalty-free basis to BMJ Publishing Group Ltd ("BMJ") its licensees and where the relevant Journal is co-owned by BMJ to the co-owners of the Journal, to publish the Work in this journal and any other BMJ products and to exploit all rights, as set out in our licence.

The Submitting Author accepts and understands that any supply made under these terms is made by BMJ to the Submitting Author unless you are acting as an employee on behalf of your employer or a postgraduate student of an affiliated institution which is paying any applicable article publishing charge ("APC") for Open Access articles. Where the Submitting Author wishes to make the Work available on an Open Access basis (and intends to pay the relevant APC), the terms of reuse of such Open Access shall be governed by a Creative Commons licence – details of these licences and which Creative Commons licence will apply to this Work are set out in our licence referred to above.

Other than as permitted in any relevant BMJ Author's Self Archiving Policies, I confirm this Work has not been accepted for publication elsewhere, is not being considered for publication elsewhere and does not duplicate material already published. I confirm all authors consent to publication of this Work and authorise the granting of this licence.

For peer review only

Fertile women's lived experiences of life before and after bariatric surgery: An interpretative qualitative study

Rebecca Paul [a,b,c], Ellen Andersson [a], Torsten Olbers [a,d], Jessica Frisk [a], Carina Berterö [e]

[a] Department of Surgery and Department of Biomedical and Clinical Sciences, Linköping University, Norrköping 60379, Sweden.

[b] Department of Surgery, Falun County Hospital, Falun 79182, Sweden.

[c] Center for Clinical Research Dalarna-Uppsala University, Sweden.

[d] Wallenberg Centre for Molecular Medicine, Department of Biomedical and Clinical Sciences, Linköping University 58185, Sweden.

[e] Division of Nursing Sciences and Reproductive Health, Department of Health, Medicine and Caring Sciences, Linköping University, Linköping 58185, Sweden.

Correspondence to**:**

Rebecca Paul, Department of Surgery, Falu Lasarett, Lasarettsvägen 10, 79182. Telephone: +46 (0)737561693. Email: rebecca.paul@liu.se. https://orcid.org/0000-0001-5355-8276.

Word count: 4109 words

Keywords: Bariatric surgery, Life change event, Obesity, Hermeneutics, Women's health.

For peer review only

ABSTRACT

Objectives: The study aimed to explore the lived experiences of women with severe obesity before and after undergoing bariatric surgery with a special focus on possible effects of changed sex hormone levels.

Design: A qualitative interview study with transcribed text analysis based on Gadamer's hermeneutics.

Setting: Regional hospital and outpatient bariatric clinic in central Sweden.

Participants: Ten women (age 23-38 years) having undergone Roux-en-Y gastric bypass surgery between 2016-2019 were interviewed.

Results: The transcribed interviews were analyzed according to Gadamer's hermeneutics. Text horizons, interpreter horizons and fact horizons were derived and formed the fusions "Recognition of unhealthy body weight", "Dealing with other people's opinions and society's norms", "Life has changed in a positive way" and "Accepting inner self and bodily changes".

Conclusion: Women highlighted weight and body size in their responses. The study provided a deeper understanding of the situation of women living with obesity and pros and cons of having undergone bariatric surgery. Experiences of changes in sex hormones and fertility were discussed but not central to the informants. Participants emphasized the need of being prepared and properly supported in dealing with changes in life after bariatric surgery and subsequent weight loss.

For peer review only

ARTICLE SUMMARY: (Strengths and limitations of this study)

- Interview study that allows for an in-depth understanding of the explored situation.
- Gadamer's hermeneutics allows for an interpretation of the rich text data with consideration taken to the interpreter's pre-understanding as well as current facts related to the phenomena presented by the interviewed women.
- The interviewed women prioritized a focus on weight and body size and the descriptions concerning experiences of changed hormones were not as emphasized as expected by the research team despite questions concerning hormones.
- The use of hormone-based contraceptives may have been a confounding factor influencing experiences in changes of hormones in some women.

For peer review only

INTRODUCTION

Obesity is a global health issue that has reached epidemic proportions. In 2017, the World Health Organization reported that overweight and obesity led to 4 million deaths worldwide (1). Obesity is associated with an increased risk of developing comorbidities that can lower quality of life, decrease physical function, and shorten life span. Medical consequences of having obesity include cardiovascular diseases, diabetes, musculoskeletal disorders, certain forms of cancer and effects on sex hormone regulation and fertility (2, 3).

Altered sex hormone regulation in women with obesity can lead to subfertility, perinatal complications, as well as bodily changes secondary to hyperandrogenism (4, 5). Effects of obesity on fecundity are oligo-/anovulation, reduced number of viable oocytes and disrupted implantation (6). Pregnant women with obesity are at increased risk of miscarriage, gestational diabetes and pre-eclampsia as well as large for gestational age babies, congenital defects and birth complications such as hemorrhage (7).

The disrupted female sex hormone balance leads to male-pattern fat dispersion with central fat accumulation, as well as hirsutism with increased body hair, especially in the facial area. Theses hormonal consequences lead to the loss of female body form and in conjunction with amenorrhea and subfertility lead to an experience of lost female characteristics (8). These can all have effects on women's physical and psychosocial health and well-being (9).

Bariatric surgery, such as Roux-en-Y gastric bypass surgery (RYGB), is a well-proven treatment leading to sustained weight loss and improvements in comorbidities in patients with severe obesity (10). Previous studies have found a reversal of disrupted sex hormone levels in women and improved fertility after RYGB (11, 12). Other studies show that women experience several physical changes after bariatric surgery with a return to a more female pattern of fat and hair dispersion (13, 14).

For peer review only

Several quantitative studies utilizing questionnaires have been conducted to investigate health and quality of life after bariatric surgery. Results suggest that physical components of quality of life improve and remain so over time after bariatric surgery while short term improvements in mental components of quality of life during the first year are followed by a long-term erosion to preoperative levels (15, 16). Data on mental health (i.e., depression, anxiety, self-harm) after bariatric surgery are conflicting (17). However, although high BMI-levels are associated with an increased prevalence of mental health challenges there does not seem to be any long-term covariation between mental health and weight loss (18).

Qualitative studies have investigated the experiences of patients living with obesity (19, 20) and after undergoing bariatric surgery (21, 22). However, to our knowledge, no qualitative studies have been conducted to explore women's encounters of hormonal changes after bariatric surgery.

This qualitative study aims to explore the lived experiences of fertile women with obesity from prior to and after undergoing laparoscopic Roux-en-Y gastric bypass surgery with an intended focus on experiences related to hormonal changes after surgery.

METHODS

Gadamer's hermeneutic analysis was chosen in order to achieve a richer and deeper understanding of the experiences described by the women included in the study (23). Creating a whole broader meaning out of individual narratives while taking into consideration the pre-understanding of the interpreter and the current accepted fact of the time is a hallmark of hermeneutic technique.

Sample

Purposive sampling strategy was utilized and women considered to be of fertile age, 18-40 years, that had previously undergone Roux-en-Y gastric bypass surgery between 2016-2019

For peer review only

were the focus of the study sample. The time interval of surgery was chosen for a recent memory of life before surgery while allowing for experience of changes after surgery. Exclusion factors were women who do not speak Swedish or were otherwise prevented from participating in an interview such as difficulty in understanding questions or expressing themselves. Thirty women fitting the inclusion and exclusion criteria were identified from the bariatric surgery outpatient clinic in Falun County Sweden, and contact was made by sending information per post indicating that the first author would be communicating with the women within a few weeks to consider participation in the study. Women were provided a telephone number and an e-mail to contact in case they desired to opt out and not want any further contact. Study participant information containing a consent form was included in the posted information. Twelve women initially accepted participation. However, 2 women later declined due to family illnesses and 10 women (age 23-38 years) were subsequently interviewed. A number of ten was deemed to be an appropriate sample size due to the interpretative qualitative method used in this study (24).

Interview

Participants were allowed to choose the environment that they found most comfortable to be interviewed in (6 in the home, 1 at hospital conference room, 1 at local library study room, 2 per telephone). Interviews were carried out by a single trained qualitative researcher (RP) as conversations with support of an interview guide with open-ended questions. The interview guide was based on the study aim although open-ended and general terms were utilized to avoid influencing the women's responses (table 1). Interviews were recorded using a digital pocket recorder and transcribed verbatim using a word processing program. The interviews lasted a median of 50 minutes, range 32-62 minutes. Interview transcripts were reviewed, and interview techniques discussed between the interviewer (RP) and a qualitative researcher (CB).

For peer review only

Table 1. Interview Guide (original language: Swedish).

1) Tell us how your life has changed since undergoing bariatric surgery? (Social, individual)
2) Tell me, how has your body changed since the surgery? (Appearance, function, experience)
3) Tell me how your body functions since the surgery? (Weight-wise, stamina, size, hormonal)
4) How do you feel that hormones affect your body? (Hormones)
Follow-up questions:<br>a) Can you tell us more about this?<br>b) How has this affected you?<br>c) Can you elaborate on this?<br>d) Tell me more….<br>e) How has this turned out since the surgery?

Analysis

The four principles of Gadamer's hermeneutic analysis (23, 25) were used during data analysis and interpretation: 1) Data immersion was carried out by reading through the transcripts repeatedly creating an understanding of the text as a whole. 2) An analysis of the text was carried out with meaningful excerpts of text from the transcripts being extracted and discussed (text horizon) 3) The interpreter's translation and understanding of the text excerpt was described and deliberated (interpreter horizon) and thereafter current facts about the existing meaningful text were presented (fact horizon). 4) Horizons were merged into fusions

For peer review only

presented in a whole text providing a broader and richer understanding of the phenomena exposed from the women's experiences. The whole study proceeded as a hermeneutic circle, from the part to the whole and back again, woman by woman, sentence by sentence. See table 2 for an example of analysis.

The first and the last author (RP and CB respectively) read the transcripts and had a continuous dialogue and review throughout the analysis process.

Table 2. Example of data analysis according to Gadamer's hermeneutics.

Horizon of text (participant)	Horizon of researcher	Horizon of literature/facts	Comments	Fusion of the horizons
Yes, eeeeh, physically of course, I've lost 50 kilos, it's nothing that I've noticed, like, it's when you em, partly it's when I buy new clothes, but I still don't really understand, I think I look about the same now as I did then.	*Not aware that looks have changed concerning weight loss<br><br>*Weight loss becomes tangible by buying clothing	*Increased body dysmorphia and "ghost fat" in the time period directly after surgery	*Mind and body conscientiousness<br><br>*Body awareness after weight loss<br><br>*Takes time before it becomes clear that body and appearance have changed and it was through buying clothing that it became more obvious.	**Accepting inner self and bodily changes**

For peer review only

Patients and public involvement statement

Patients were not directly involved in the planning, design or recruiting process of the study. Previous concerns posed by patients in clinical experience and previous studies (methods of recruitment, sensitivity to having obesity, comfortable interview-situation) were taken into account in the development of the project design. The results will be shared with the patients after acceptance with an e-mailed summary of the study.

RESULTS

The analysis performed with Gadamer's hermeneutics, using the different horizons referring to the integration of constructions given by patients, by our interpretation of situations explained in interviews, by our professional background and by selected literature made it possible to merge the fusions "Recognition of unhealthy body weight", "Dealing with other people's opinions and society's norms", "Life has changed in a positive way" and "Accepting inner self and bodily changes" (see Figure 1).

Recognition of unhealthy body weight

The women expressed a realization that they previously were living with an unhealthy weight and needed to make the necessary decisions to do something to change their situation and reduce the risks of comorbidities related to obesity. They felt a hopelessness in that their weight always ended up increasing after dieting and that they often regained more weight than was lost. This inability to maintain a healthy weight after repeated weight loss led to feelings of shame and ineptitude where they felt that they were always blamed for having obesity.

> *I had a very hard time losing weight on my own, I tried a lot of different things, and it was hard, I worked out, ate GI, did weight watchers, everything, and only lost a few pounds, and then when I stopped it, I went up the double. (W1)*

> *It was mostly disappointment and anger at myself like how I could have done this to myself, how could I do this to my body. (W4)*

The women described joint, back and foot pain as well as constant exhaustion from carrying the extra weight that kept them from exercising, hindered them from taking part in employment and isolated them from the outside world. Their health problems became evident when they developed co-morbidities to obesity and these consequences made it clear to them that having obesity was a threat to their health and that they needed to seek help for a more effective weight loss.

For peer review only

It felt terrible, so I was sad, really, I felt bad like…. you realized that this is not good either and I was not old, I was 25 years old and could not run up a hill. (W3)

The women feared that adverse effects on health from having obesity would threaten their abilities to parent by shortening their life span and diminishing capability to be physically active with their children.

Now my excess weight will eventually affect my heart and I have a child who was 4 at the time. Who will take care of her if I have a heart attack or stroke and die and disappear? I want to see her grow up and that's what made me do the surgery, that was the last straw. (W2)

But it was also that the children said, "but mother why are you so tired, why don't you want to do anything, and you are boring, and you never want to do anything", and you heard it all the time. (W4)

Several of the women described difficulties in becoming pregnant before surgery and that they were aware that having obesity could lead to subfertility and reproduction-related difficulties. They feared that having obesity could hinder them from being able to have children of their own.

We tried for several years actively (to conceive) and it did not work, by itself, so we had to seek help in the end. However, I knew that the first thing they will say is that you both need to lose weight. (W5)

Dealing with other people's opinions and society norms

The women expressed how dealing with other people's opinions of them and their weight affected the way that they felt about themselves. They did not want to be around other people

For peer review only

and they felt that the stigma associated with being overweight made them less valued. The lack of knowledge concerning the causes of obesity among the public led the women to feel shame for their situation, weight, and appearance.

I thought that nothing was fun when I was big, I didn't want to go out, I didn't want to meet people, I kept to myself a lot, because I felt that no, I had no business being around others, I didn't feel nice enough to be around other people. (W2)

I have knowledge about my operation, about me, so it's probably very difficult for others who haven't had to be in this, in what it is to have obesity, and how it is to have surgery, and the knowledge is very low among others…including healthcare personnel. (W8)

Many of the women presented their experiences of being singled out for having obesity. The women felt shame and worthlessness from their body size and evaded unwanted attention from others because they felt that the norms of society determined a person's value based on how attractive they are perceived to be in a social group.

It was mostly that I had the feeling that I was being watched when I was out among people, and whether I was in a galleria or in town, just going out and having a coffee with a friend, I felt that people were staring at me because I was so big, uh, and therefore, I withdrew to stay away from being among people. (W4)

Being fat is ugly, you've been taught that since you were little by society, that you're outside the norm, and that's probably what's ingrained in your mind, that you're a little less worthy, so to speak. (W5)

For peer review only

Social media's influence on stigma was expressed repeatedly by the interviewed women. The harsh and mean-spirited comments from anonymous persons could have serious effects on the women's self-esteem and mental health and led to them feeling even more isolated from society.

> *Post a picture on social media, and people comment on whether you looked good or not...I think there was a lot of cyberbullying, like back then, if you didn't look good you were told so.... a lot of that started then...it probably started in my generation. (W7)*

The women feared that media's influence on body image could affect the way their children perceived themselves or were treated and they expressed a need for more knowledge concerning obesity and bariatric surgery in order to reduce stereotypes.

> *I think that it's hard to change with people, actually, I can see as well, um, one of my children, who is not at all overweight, at all, has expressed now quite recently that she's not comfortable with her body, and that she feels fat. (W8)*

Life has changed in a positive way

The women that were interviewed expressed that surgery led to their lives being changed in a positive way. The decreased weight burden led to increased energy and improved mobility with greater endurance for physical activity which allowed for more healthy lifestyles as well as feeling more motivated to be socially active.

> *I think I'm happier as a person, more grateful, I'm trying to, like, take advantage of life, I'm going to, like, take care of myself more with both diet and exercise, I'm grateful that I got this chance. (W1)*

For peer review only

Improved endurance and mobility with reduced musculoskeletal pain allowed the women to be employable again and improved their economic situation, including potential for career advancement. Often, there had been several failed attempts previously to rehabilitate to work due to exhaustion and physical pain.

> *It made a big difference at the workplace because I had back pain. That is the most important thing about having the operation, is that I can live a life again. (W9)*

Family life and relationships with their children were enriched after the weight loss from surgery. The women talked about how increased energy and mobility made it possible for them to be the parents they envisioned by playing with their children and taking part in games, outdoor activities and visits to amusement parks without fear of difficulties from their body size.

> *Everything has become so much easier since I got rid of the extra kilos that were there and were bothering me before. Everything has become a lot easier, yes, I was able to socialize and activate my child in a completely different way too. (W2)*

The women explained the importance of clothing and how being able to fit into the current trendy styles included them into society as well as the ability to share clothing with others bringing feelings of fellowship and solidarity. The reduced clothing size after surgery was a reminder of their successful weight loss and a motivator to continue their healthy lifestyles.

> *It is so clear that it is a boost, that you can choose more clothing and any clothing store, that you are part of society.*

> *You don't feel like the elephant in the room anymore, you can go to a normal store and buy clothes, so it's changed a lot. (W7)*

For peer review only

The women described how male-pattern fat dispersion dissipated after surgery and that they experienced more distinct female contours with a defined waist and hips. They felt more confident having more feminine characteristics and enjoyed that clothing fit their figures in a more complementary way.

> *At my heaviest my body shape very square, and no waist and not very much shape at all, hips and shoulders, everything was about the same width, and that's something that I noticed…. that I had more defined body parts, so it's a change, purely in terms of appearance. (W6)*

Sex lives were improved after weight loss and the women explained how the increase in energy, reduced body size and enhanced self-confidence increased their sex drive and motivation for taking part in sexual activities. The women described that they no longer found it uncomfortable to show their bodies without clothing.

> *I get turned on to things more easily, it's not the same, before it was like I went back and thought no... I don't want to...so there have been some changes now, that the body reacts differently than what it did before...and it has, it's nice in a way, because before I thought there was something wrong with me. (W7)*

Accepting inner self and bodily changes

The women experienced many physical alterations after surgery and felt it was difficult to understand, adjust to and accept everything that occurred which led to a feeling of their mind not keeping up with the physical changes in their bodies. They felt that they were still the same size as before their surgery and were often only reminded when they purchased clothing and had to buy much smaller sizes.

For peer review only

The brain doesn't react with what's happening, you don't stand in front of the mirror all the time and keep track, at least not me, the brain just doesn't keep up, that everything changes. (W9)

The head did not keep up with the body, so it takes longer than the body to keep up with the journey. (W4)

It was important for the interviewed women to maintain the same personality and way of thinking as before the surgery with the importance of not allowing weight loss and a changed exterior to influence their opinions of themselves. The women reacted strongly to the more positive treatment they experienced based on their looks since they felt they were the same person on the inside.

I have the attitude that weight has nothing to do with who I am, it has nothing to do with me, I'm still the same person whether I weigh so much or so little. (W10)

There were side effects of surgery that the women dealt with in different ways. The resulting excess skin from decreased fat tissue reminded the women of their previous weight, contributed to a continued large body size, and led to certain hinders in finding clothing that fit properly and at times difficulty with hygiene. The women talked about a tolerance for dumping symptoms and early satiety and accepted that it was part of the effectiveness of the surgery.

You look like you weigh more than you do because you have so much excess skin. (W1)

I get heart palpitations sometimes, so you are physically reminded that you have been careless with food, so it is a huge change that the body does not react to food in the same way at all as before the operation. Now it reacts when you eat

For peer review only

too much, or you can feel a little like maybe you shouldn't have eaten that type of food. (W8)

DISCUSSION

This study was designed to explore the lived experiences of women with obesity before and after undergoing bariatric surgery and possible effects of changed sex hormone levels. The principal findings reflected the recognition of an unhealthy body weight as a motivation for surgery and that dealing with society's norms and other people's opinions had a profound effect on their quality of life and mental health before surgery. The women also described the changes after surgery that had a positive influence on their daily lives as well as challenges in adapting mentally to the physical alterations after weight loss. Although hormonal changes after surgery were not a central focus, the women talked about related physical alterations that could be interpreted as hormonal effects that became a part of the whole experience.

Prior to surgery, adverse social experiences based on body size led to isolation and avoidance of social contacts potentially increasing the risk of developing mental health issues, such as depression and anxiety (26). Stigmatizing attitudes in society have been found in previous studies to have a basis in a lack of knowledge about the pathogenesis of obesity, as well as believing that obesity is associated with negative stereotypes such as laziness, lower intelligence, lack of motivation and self-control (27). The existence of stigma is even present within the healthcare field and can lead to the women receiving substandard care (28).

Clothing size symbolized success and being able to wear and share trendy and standard sized garments made women feel included in a larger context. Work apparel was another aspect representing inability to "fit in". A previous study suggested that smaller clothing sizes signified a healthy body weight and associated with more positive self-esteem and improved body image (29). Clothing size has also been used in previous studies as a measure of risk for

For peer review only

developing comorbidities (30, 31). The women stated that before surgery, their larger attire was a reminder of their unhealthy weight and that reaching the desired weight and clothing size symbolized their journey from having obesity to obtaining a healthy body.

The rapid weight loss and development of excess skin after surgery led to the "mind not following the body" corroborating previous research that reported difficulties adjusting to the new body appearance and resulting in a discordance in their identity perception (32). Interestingly, research has shown that patients who postoperatively identified themselves as non-obese were more satisfied with their body size than those patients who had lost weight but still identified themselves as having obesity. Fortunately, women became more satisfied with their body size over time (33). In a study by Gilmartin women who had undergone bariatric surgery emphasized difficulties in changes in body image and a need for support (34). A meta-analysis showed difficulties in finding standard follow-up measures that take into account all factors that influence a patient's satisfaction with their body after surgery (35). Preoperative information about expected changes and experiences of others, and support postoperatively may enable women to cope with the "mind not following the body".

Quantitative studies have shown restoration of sex hormone regulation in women after bariatric surgery (36) and experiences likely secondary to that were expressed by several women in this study. Satisfaction with having a defined waist, reduction of central fat dispersion as well as restitution of menstrual cycles, more profound cyclic variations in mood after surgery and better sex life were expressed. This was associated with a concrete feeling of success with surgery and a reinforcement of the health effects of weight loss on the body's function. Lack of awareness about the relationship between sex hormones and experienced symptoms may, however, have led to not catching certain aspects of hormonal changes during the interviews.

For peer review only

In conclusion, this study of women´s lived experiences before and after bariatric surgery provided information suggesting that issues related to changes in body size and other people´s attitudes are dominating. Experiences that could be related to changes in sex hormones were mentioned, but not central in the women´s narratives. Findings can preferably be included in the information to women seeking bariatric surgery and be repeated as a routine during follow-up.

Acknowledgments

We look like to extend our gratitude to the bariatric clinic in Falun for assisting in the recruitment of the study participants.

Author contributions

R.P conceived the idea of conducting the study with support and supervision from C.B., T.O., J.F. and E.A. The theory, methodology and interview guide were discussed by all authors and determined with expert supervision from C.B. R.P. performed the interviews and the transcription and discussed and processed the data under supervision from C.B. Analysis was carried out by R.P. and C.B. R.P drafted the manuscript which all authors revised into the final manuscript.

Funding

This work was supported by grant number 06000984 from the County Council of Östergötland and grant number 974932 from the County Council of Dalarna, Sweden.

Competing Interests

TO participated in advisory boards and educational activities for Johnson&Johnson and Novo Nordisk unrelated to the submitted article, and reimbursements were directed to his academic institution. All other authors have no competing interests to declare.

Data sharing information

For peer review only

Anonymised data will be made available upon request made to the corresponding author.

Ethics Approval Statement

The study was approved in April 2022 by the Swedish Ethical Review Authority, case number 2022-01708-01. All procedures were in accordance with the ethical standards of the 1964 Helsinki declaration and its later amendments. Written and verbal informed consent was obtained from all study participants. Close consideration was taken to protect identities due to the sensitive subject and the limited number of informants.

Figure 1. Visualize the fusions and their relationship.

For peer review only

REFERENCES

1. World Health Organization. Obesity and overweight. 2017.

2. Guh DP, Zhang W, Bansback N, et al. The incidence of co-morbidities related to obesity and overweight: a systematic review and meta-analysis. BMC public health. 2009;9:88.

3. Balen AH, Anderson RA. Impact of obesity on female reproductive health: British Fertility Society, Policy and Practice Guidelines. Human fertility (Cambridge, England). 2007;10(4):195-206.

4. Brewer CJ, Balen AH. The adverse effects of obesity on conception and implantation. Reproduction (Cambridge, England). 2010;140(3):347-64.

5. Wei S, Schmidt MD, Dwyer T, et al. Obesity and menstrual irregularity: associations with SHBG, testosterone, and insulin. Obesity (Silver Spring, Md). 2009;17(5):1070-6.

6. Broughton DE, Moley KH. Obesity and female infertility: potential mediators of obesity's impact. Fertility and sterility. 2017;107(4):840-7.

7. Catalano PM, Shankar K. Obesity and pregnancy: mechanisms of short term and long term adverse consequences for mother and child. BMJ (Clinical research ed). 2017;356:j1.

8. Silvestris E, de Pergola G, Rosania R, et al. Obesity as disruptor of the female fertility. Reproductive biology and endocrinology : RB&E. 2018;16(1):22.

9. Chu DT, Minh Nguyet NT, Nga VT, et al. An update on obesity: Mental consequences and psychological interventions. Diabetes & metabolic syndrome. 2019;13(1):155-60.

10. Adams TD, Davidson LE, Litwin SE, et al. Weight and Metabolic Outcomes 12 Years after Gastric Bypass. The New England journal of medicine. 2017;377(12):1143-55.

11. Sarwer DB, Spitzer JC, Wadden TA, et al. Changes in sexual functioning and sex hormone levels in women following bariatric surgery. JAMA surgery. 2014;149(1):26-33.

12. Paul R, Andersson E, Wirén M, et al. Health-Related Quality of Life, Sexuality and Hormone Status after Laparoscopic Roux-En-Y Gastric Bypass in Women. Obesity surgery. 2020;30(2):493-500.

13. Li YJ, Han Y, He B. Effects of bariatric surgery on obese polycystic ovary syndrome: a systematic review and meta-analysis. Surgery for obesity and related diseases : official journal of the American Society for Bariatric Surgery. 2019;15(6):942-50.

14. Skubleny D, Switzer NJ, Gill RS, et al. The Impact of Bariatric Surgery on Polycystic Ovary Syndrome: a Systematic Review and Meta-analysis. Obesity surgery. 2016;26(1):169-76.

For peer review only - http://bmjopen.bmj.com/site/about/guidelines.xhtml

15. Kolotkin RL, Andersen JR. A systematic review of reviews: exploring the relationship between obesity, weight loss and health-related quality of life. Clinical obesity. 2017;7(5):273-89.

16. Jumbe S, Bartlett C, Jumbe SL, et al. The effectiveness of bariatric surgery on long term psychosocial quality of life - A systematic review. Obesity research & clinical practice. 2016;10(3):225-42.

17. Dawes AJ, Maggard-Gibbons M, Maher AR, et al. Mental Health Conditions Among Patients Seeking and Undergoing Bariatric Surgery: A Meta-analysis. Jama. 2016;315(2):150-63.

18. Silva DA, Coutinho E, Ferriani LO, et al. Depression subtypes and obesity in adults: A systematic review and meta-analysis. Obesity reviews : an official journal of the International Association for the Study of Obesity. 2020;21(3):e12966.

19. Ogden K, Barr J, Rossetto G, et al. A "messy ball of wool": a qualitative study of the dimensions of the lived experience of obesity. BMC psychology. 2020;8(1):67.

20. Haga BM, Furnes B, Dysvik E, et al. Putting life on hold: lived experiences of people with obesity. Scandinavian journal of caring sciences. 2020;34(2):514-23.

21. Coulman KD, MacKichan F. Patients' experiences of life after bariatric surgery and follow-up care: a qualitative study. 2020;10(2):e035013.

22. Lier H, Aastrom S, Rørtveit K. Patients' daily life experiences five years after gastric bypass surgery--a qualitative study. Journal of clinical nursing. 2016;25(3-4):322-31.

23. Gadamer H-G. Truth and method: A&C Black; 2013.

24. Kvale S, Brinkmann S. InterViews : learning the craft of qualitative research interviewing. Los Angeles: Sage Publications; 2009.

25. Patton MQ. Qualitative Research & Evaluation Methods: integrating theory and practice. Thousand Oaks, CA.: SAGE Publications, Inc.; 2015.

26. Sarwer DB, Fabricatore AN, Eisenberg MH, et al. Self-reported stigmatization among candidates for bariatric surgery. Obesity (Silver Spring, Md). 2008;16 Suppl 2:S75-9.

27. Friedman KE, Ashmore JA, Applegate KL. Recent experiences of weight-based stigmatization in a weight loss surgery population: psychological and behavioral correlates. Obesity (Silver Spring, Md). 2008;16 Suppl 2:S69-74.

28. Phelan SM, Burgess DJ, Yeazel MW, et al. Impact of weight bias and stigma on quality of care and outcomes for patients with obesity. Obesity reviews : an official journal of the International Association for the Study of Obesity. 2015;16(4):319-26.

For peer review only

29. Petroff AB, Martz DM, Webb RM, et al. Predicting ideal body mass index: what does clothing size have to do with it? Body image. 2011;8(2):126-34.
30. Hughes LA, Schouten LJ, Goldbohm RA, et al. Self-reported clothing size as a proxy measure for body size. Epidemiology (Cambridge, Mass). 2009;20(5):673-6.
31. Han TS, Gates E, Truscott E, et al. Clothing size as an indicator of adiposity, ischaemic heart disease and cardiovascular risks. Journal of human nutrition and dietetics : the official journal of the British Dietetic Association. 2005;18(6):423-30.
32. Souza ALL, de Souza PM, Mota BEF, et al. Ghost Fat: Altered Female Body Perception After Bariatric Surgery. Perceptual and motor skills. 2022:315125221133868.
33. Perdue TO, Schreier A, Swanson M, et al. Evolving self view and body image concerns in female postoperative bariatric surgery patients. Journal of clinical nursing. 2018;27(21-22):4018-27.
34. Gilmartin J. Body image concerns amongst massive weight loss patients. Journal of clinical nursing. 2013;22(9-10):1299-309.
35. Ivezaj V, Grilo CM. The complexity of body image following bariatric surgery: a systematic review of the literature. Obesity reviews : an official journal of the International Association for the Study of Obesity. 2018;19(8):1116-40.
36. Sarwer DB, Wadden TA, Spitzer JC, et al. 4-Year Changes in Sex Hormones, Sexual Functioning, and Psychosocial Status in Women Who Underwent Bariatric Surgery. Obesity surgery. 2018;28(4):892-9.

For peer review only

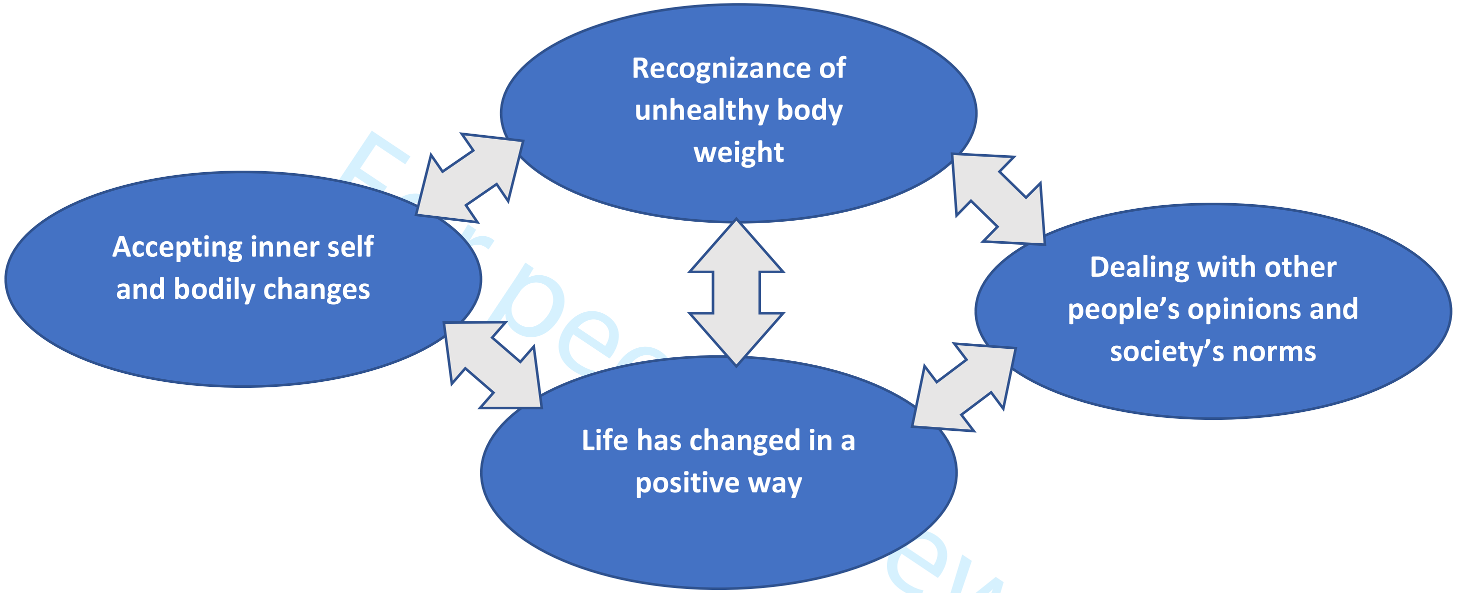

For peer review only

COREQ (COnsolidated criteria for REporting Qualitative research) Checklist

A checklist of items that should be included in reports of qualitative research. You must report the page number in your manuscript where you consider each of the items listed in this checklist. If you have not included this information, either revise your manuscript accordingly before submitting or note N/A.

Topic	Item No.	Guide Questions/Description	Reported on Page No.
**Domain 1: Research team and reflexivity**			
Personal characteristics			
Interviewer/facilitator	1	Which author/s conducted the interview or focus group?	
Credentials	2	What were the researcher's credentials? E.g. PhD, MD	
Occupation	3	What was their occupation at the time of the study?	
Gender	4	Was the researcher male or female?	
Experience and training	5	What experience or training did the researcher have?	
Relationship with participants			
Relationship established	6	Was a relationship established prior to study commencement?	
Participant knowledge of the interviewer	7	What did the participants know about the researcher? e.g. personal goals, reasons for doing the research	
Interviewer characteristics	8	What characteristics were reported about the inter viewer/facilitator? e.g. Bias, assumptions, reasons and interests in the research topic	
**Domain 2: Study design**			
Theoretical framework			
Methodological orientation and Theory	9	What methodological orientation was stated to underpin the study? e.g. grounded theory, discourse analysis, ethnography, phenomenology, content analysis	
Participant selection			
Sampling	10	How were participants selected? e.g. purposive, convenience, consecutive, snowball	
Method of approach	11	How were participants approached? e.g. face-to-face, telephone, mail, email	
Sample size	12	How many participants were in the study?	
Non-participation	13	How many people refused to participate or dropped out? Reasons?	
Setting			
Setting of data collection	14	Where was the data collected? e.g. home, clinic, workplace	
Presence of non-participants	15	Was anyone else present besides the participants and researchers?	
Description of sample	16	What are the important characteristics of the sample? e.g. demographic data, date	
Data collection			
Interview guide	17	Were questions, prompts, guides provided by the authors? Was it pilot tested?	
Repeat interviews	18	Were repeat inter views carried out? If yes, how many?	
Audio/visual recording	19	Did the research use audio or visual recording to collect the data?	
Field notes	20	Were field notes made during and/or after the inter view or focus group?	
Duration	21	What was the duration of the inter views or focus group?	
Data saturation	22	Was data saturation discussed?	
Transcripts returned	23	Were transcripts returned to participants for comment and/or	

For peer review only

For peer review only - http://bmjopen.bmj.com/site/about/guidelines.xhtml

Topic	Item No.	Guide Questions/Description	Reported on Page No.
		correction?	
**Domain 3: analysis and findings**			
Data analysis			
Number of data coders	24	How many data coders coded the data?	
Description of the coding tree	25	Did authors provide a description of the coding tree?	
Derivation of themes	26	Were themes identified in advance or derived from the data?	
Software	27	What software, if applicable, was used to manage the data?	
Participant checking	28	Did participants provide feedback on the findings?	
Reporting			
Quotations presented	29	Were participant quotations presented to illustrate the themes/findings? Was each quotation identified? e.g. participant number	
Data and findings consistent	30	Was there consistency between the data presented and the findings?	
Clarity of major themes	31	Were major themes clearly presented in the findings?	
Clarity of minor themes	32	Is there a description of diverse cases or discussion of minor themes?	

Developed from: Tong A, Sainsbury P, Craig J. Consolidated criteria for reporting qualitative research (COREQ): a 32-item checklist for interviews and focus groups. *International Journal for Quality in Health Care*. 2007. Volume 19, Number 6: pp. 349 – 357

Once you have completed this checklist, please save a copy and upload it as part of your submission. DO NOT include this checklist as part of the main manuscript document. It must be uploaded as a separate file.

For peer review only

BMJ Open

Women's lived experiences of sex hormones and life related to bariatric surgery: An interpretative qualitative study

Journal:	*BMJ Open*
Manuscript ID	bmjopen-2023-072085.R1
Article Type:	Original research
Date Submitted by the Author:	30-May-2023
Complete List of Authors:	Paul, Rebecca; Linköping University, Department of Surgery Department of Biomedical and Clinical Sciences; Center for Clinical Research Dalarna, Department of Surgery, Falun County Hospital<br>Andersson, Ellen; Linköping University, Department of Surgery and Department of Biomedical and Clinical Sciences<br>Olbers, Torsten ; Linköping University, Department of Surgery and Department of Biomedical and Clinical Sciences; Linköping University, Wallenberg Centre for Molecular Medicine, Department of Biomedical and Clinical Sciences<br>Frisk, Jessica; Linköping University, Department of Surgery and Department of Biomedical and Clinical Sciences<br>Berterö, Carina; Linköping University, Division of Nursing Sciences and Reproductive Health, Institution of Medical and Health Sciences
**Primary Subject Heading**:	Qualitative research
Secondary Subject Heading:	Surgery, Sexual health, Nutrition and metabolism, Diabetes and endocrinology, Reproductive medicine
Keywords:	Bariatric Surgery, Life Change Events, Obesity, Hermeneutics, Sex steroids & HRT < DIABETES & ENDOCRINOLOGY

SCHOLARONE™
Manuscripts

I, the Submitting Author has the right to grant and does grant on behalf of all authors of the Work (as defined in the below author licence), an exclusive licence and/or a non-exclusive licence for contributions from authors who are: i) UK Crown employees; ii) where BMJ has agreed a CC-BY licence shall apply, and/or iii) in accordance with the terms applicable for US Federal Government officers or employees acting as part of their official duties; on a worldwide, perpetual, irrevocable, royalty-free basis to BMJ Publishing Group Ltd ("BMJ") its licensees and where the relevant Journal is co-owned by BMJ to the co-owners of the Journal, to publish the Work in this journal and any other BMJ products and to exploit all rights, as set out in our licence.

The Submitting Author accepts and understands that any supply made under these terms is made by BMJ to the Submitting Author unless you are acting as an employee on behalf of your employer or a postgraduate student of an affiliated institution which is paying any applicable article publishing charge ("APC") for Open Access articles. Where the Submitting Author wishes to make the Work available on an Open Access basis (and intends to pay the relevant APC), the terms of reuse of such Open Access shall be governed by a Creative Commons licence – details of these licences and which Creative Commons licence will apply to this Work are set out in our licence referred to above.

Other than as permitted in any relevant BMJ Author's Self Archiving Policies, I confirm this Work has not been accepted for publication elsewhere, is not being considered for publication elsewhere and does not duplicate material already published. I confirm all authors consent to publication of this Work and authorise the granting of this licence.

For peer review only

Women's lived experiences of sex hormones and life related to bariatric surgery: An interpretative qualitative study

Rebecca Paul [a,b,c], Ellen Andersson [a], Torsten Olbers [a,d], Jessica Frisk [a], Carina Berterö [e]

[a] Department of Surgery and Department of Biomedical and Clinical Sciences, Linköping University, Norrköping 60379, Sweden.

[b] Department of Surgery, Falun County Hospital, Falun 79182, Sweden.

[c] Center for Clinical Research Dalarna-Uppsala University, Sweden.

[d] Wallenberg Centre for Molecular Medicine, Department of Biomedical and Clinical Sciences, Linköping University 58185, Sweden.

[e] Division of Nursing Sciences and Reproductive Health, Department of Health, Medicine and Caring Sciences, Linköping University, Linköping 58185, Sweden.

Correspondence to:

Rebecca Paul, Department of Surgery, Falu Lasarett, Lasarettsvägen 10, 79182. Telephone: +46 (0)737561693. Email: rebecca.paul@liu.se. https://orcid.org/0000-0001-5355-8276.

Word count: 4769 words

Keywords: Bariatric surgery, Life change event, Obesity, Hermeneutics, Sex steroids

For peer review only

ABSTRACT

Objectives: The study aimed to explore the lived experiences of women with severe obesity before and after undergoing bariatric surgery with a special focus on possible effects of changed sex hormone levels.

Design: A qualitative interview study with transcribed text analysis based on Gadamer's hermeneutics.

Setting: Regional hospital and outpatient bariatric clinic in central Sweden.

Participants: Ten women (age 23-38 years) having undergone Roux-en-Y gastric bypass surgery between 2016-2019 were interviewed.

Results: The transcribed interviews were analyzed according to Gadamer's hermeneutics. Text horizons, interpreter horizons and fact horizons were derived and formed the fusions "Recognition of unhealthy body weight", "Dealing with other people's opinions and society's norms", "Life has changed in a positive way" and "Accepting inner self and bodily changes".

Conclusion: Women highlighted weight and body size in their responses. The study provided a deeper understanding of the situation of women living with obesity and pros and cons of having undergone bariatric surgery. Experiences of changes in sex hormones and fertility were discussed but not central to the informants. Participants emphasized the need to be prepared and properly supported in dealing with changes in life after bariatric surgery and subsequent weight loss.

For peer review only

ARTICLE SUMMARY: (Strengths and limitations of this study)

- Interview study that allows for an in-depth understanding of the explored situation.
- Gadamer's hermeneutics allows for an interpretation of the rich text data with consideration taken to the interpreter's pre-understanding as well as current facts related to the phenomena presented by the interviewed women.
- The interviewed women prioritized a focus on weight and body size and the descriptions concerning experiences of changed hormones were not as emphasized as expected by the research team despite specific questions concerning hormones.
- The use of hormone-based contraceptives may have been a confounding factor influencing experiences in changes of hormones in some women.
- Language limitations may have reduced the detail in the women's descriptions as only Swedish speaking participants were included in the study.

For peer review only

INTRODUCTION

Obesity is a global health issue that has reached epidemic proportions. In 2017, the World Health Organization reported that overweight and obesity led to 4 million deaths worldwide [1]. Obesity is associated with an increased risk of developing comorbidities that can lower quality of life, decrease physical function, and shorten life span. Medical consequences of having obesity include cardiovascular diseases, diabetes, musculoskeletal disorders, certain forms of cancer and effects on sex hormone regulation and fertility [2, 3].

Altered sex hormone regulation in women with obesity can lead to subfertility, perinatal complications, as well as bodily changes secondary to hyperandrogenism [4, 5]. Effects of obesity on fertility are irregular periods, reduced number of viable egg cells and disrupted implantation [6]. Pregnant women with obesity are at increased risk of miscarriage, gestational diabetes and pre-eclampsia as well as large for gestational age babies, congenital defects and birth complications such as hemorrhage [7].

The disrupted female sex hormone balance leads to male-pattern fat dispersion with central fat accumulation, as well as hirsutism with increased body hair, especially in the facial area. Theses hormonal consequences lead to the loss of female body form and, in conjunction with irregular periods and subfertility, lead to an experience of lost female characteristics [8]. A recent study [9] has illustrated that women living with obesity have reduced self-esteem and increased body image dissatisfaction perceived as due to their weight and body shape. The effects on self-esteem and body image are also associated with higher incidence of depression influencing psychosocial health and well-being.

Bariatric surgery, such as Roux-en-Y gastric bypass surgery (RYGB), is a well-proven treatment leading to sustained weight loss and improvements in comorbidities in patients with severe obesity [10]. Previous studies have found a reversal of disrupted sex hormone levels in

For peer review only

women and improved fertility after RYGB [11, 12]. Other studies show that women experience several physical changes after bariatric surgery with a return to a more female pattern of fat and hair dispersion [13, 14].

Several quantitative studies utilizing questionnaires have been conducted to investigate health and quality of life after bariatric surgery. Results suggest that physical components of quality of life improve and remain so over time after bariatric surgery while short term improvements in mental components of quality of life during the first year are followed by a long-term erosion to preoperative levels [15, 16]. Data on mental health (i.e., depression, anxiety, self-harm) after bariatric surgery are conflicting [17]. However, although high BMI-levels are associated with an increased prevalence of mental health challenges there does not seem to be any long-term improvement in mental health secondary to weight loss [18].

Qualitative studies have investigated the experiences of patients living with obesity [19, 20] and after undergoing bariatric surgery [21, 22]. However, to our knowledge, no qualitative studies have been conducted to explore women's encounters of hormonal changes after bariatric surgery.

This qualitative study aims to explore the lived experiences of fertile women with obesity from prior to and after undergoing laparoscopic Roux-en-Y gastric bypass surgery with an intended focus on experiences related to hormonal changes after surgery.

METHODS

Gadamer's hermeneutic analysis was chosen in order to achieve a richer and deeper understanding of the experiences described by the women included in the study [23]. Gadamer's hermeneutics involves creating horizons from the participant's text, the investigators interpretation of the participant's text and supportive text from current research concerning the isolated participant text excerpts. Creating a whole broader meaning out of

For peer review only

individual narratives while taking into consideration the pre-understanding of the interpreter and the current accepted fact of the time is a hallmark of hermeneutic technique. Current accepted facts involve reviewing and including relevant contemporary studies supporting the phenomena described by the participants and merging these findings into the derived fusions.

Sample

Purposive sampling strategy was utilized and women considered to be of fertile age, 18-40 years, that had previously undergone Roux-en-Y gastric bypass surgery between 2016-2019 were the focus of the study sample. The time interval of surgery was chosen for a recent memory of life before surgery while allowing for experience of changes after surgery. Exclusion factors were women who do not speak Swedish or were otherwise prevented from participating in an interview such as difficulty in understanding questions or expressing themselves. Communication via interpreter was avoided due to the risk of information distortion through a third party and the risk of questions and responses being misunderstood. Thirty women fitting the inclusion and exclusion criteria were identified from the bariatric surgery outpatient clinic in Falun County Sweden, and contact was made by sending information per post indicating that the first author would be communicating with the women within a few weeks to consider participation in the study. Women were provided a telephone number and an e-mail to contact in case they desired to opt out and not want any further contact. Study participant information containing a consent form was included in the posted information. Twelve women initially accepted participation. However, 2 women later declined due to family illnesses and 10 women (age 23-38 years) were subsequently interviewed. A number of ten was deemed to be an appropriate sample size due to the interpretative qualitative method used in this study, and as previously described by Kvale [24].

Interview

For peer review only

Participants were allowed to choose the environment that they found most comfortable to be interviewed in (6 in the home, 1 at hospital conference room, 1 at local library study room, 2 per telephone). Interviews were carried out by a single trained qualitative researcher (RP) as conversations with support of an interview guide with open-ended questions. The interview guide was based on the study aim although open-ended and general terms were utilized to avoid influencing the women's responses (table 1). Interviews were recorded using a digital pocket recorder and transcribed verbatim using a word processing program. The interviews lasted a median of 50 minutes, range 32-62 minutes. Interview transcripts were reviewed, and interview techniques discussed between the interviewer (RP) and a qualitative researcher (CB). RP and CB reviewed the transcribed text and determined that data detail and richness was sufficient to carry out analysis without further interviews.

Table 1. Interview Guide (original language: Swedish).

1) Tell us how your life has changed since undergoing bariatric surgery?<br>(Social, individual)
2) Tell me, how has your body changed since the surgery?<br>(Appearance, function, experience)
3) Tell me how your body functions since the surgery?<br>(Weight-wise, stamina, size, hormonal)
4) How do you feel that hormones affect your body?<br>(Hormones)
Follow-up questions:<br>a) Can you tell us more about this?<br>b) How has this affected you?

For peer review only

c) Can you elaborate on this?
d) Tell me more….
e) How has this turned out since the surgery?

Analysis

The four principles of Gadamer's hermeneutic analysis [23, 25] were used during data analysis and interpretation: 1) Data immersion was carried out by reading through the transcripts repeatedly creating an understanding of the text as a whole. 2) An analysis of the text was carried out with meaningful excerpts of text from the transcripts being extracted and discussed (text horizon) 3) The interpreter's translation and understanding of the text excerpt was described and deliberated through written text (interpreter horizon) and thereafter findings from current accepted studies and literature about the existing meaningful text were presented (fact horizon). 4) Horizons were merged into fusions presented in a whole text providing a broader and richer understanding of the phenomena exposed from the women's experiences. The whole study proceeded as a hermeneutic circle, from the part to the whole and back again, woman by woman, sentence by sentence. See table 2 for an example of analysis.

The first and the last author (RP and CB respectively) read the transcripts and had a continuous dialogue and review throughout the analysis process.

Table 2. Example of data analysis according to Gadamer's hermeneutics.

Horizon of text (participant)	Horizon of researcher	Horizon of literature/facts	Comments	Fusion of the horizons
Yes, eeeeh, physically of course, I've lost 50 kilos, it's nothing that I've noticed, like, it's when you em, partly it's when I buy new	*Not aware that looks have changed concerning weight loss<br><br>*Weight loss becomes	*Increased body dysmorphia and "ghost fat" in the time period directly after surgery	*Mind and body conscientiousness<br><br>*Body awareness after weight loss<br><br>*Takes time before it becomes clear that body	**Accepting inner self and bodily changes**

For peer review only

clothes, but I still don't really understand, I think I look about the same now as I did then.	tangible by buying clothing		and appearance have changed and it was through buying clothing that it became more obvious.	

Patients and public involvement statement

Patients were not directly involved in the planning, design or recruiting process of the study. Previous concerns posed by patients in clinical experience and previous studies (methods of recruitment, sensitivity to having obesity, comfortable interview-situation) were taken into account in the development of the project design [12, 26]. The results will be shared with the patients after acceptance with an e-mailed summary of the study.

RESULTS

The analysis was performed with Gadamer's hermeneutics involving integration of the text horizon (text constructions given by patients), interpreter horizons (our interpretation of situations explained in interviews) and fact horizons (based on current selected literature). Merging of the horizons created the fusions "Recognition of unhealthy body weight", "Dealing with other people's opinions and society's norms", "Life has changed in a positive way" and "Accepting inner self and bodily changes" (see Figure 1).

Recognition of unhealthy body weight

The women expressed a realization that they previously were living with an unhealthy weight and needed to make the necessary decisions to do something to change their situation and reduce the risks of comorbidities related to obesity. They felt hopelessness in that their weight always ended up increasing after dieting and that they often regained more weight than

For peer review only

was lost. This inability to maintain a healthy weight after repeated weight loss led to feelings of shame and ineptitude where they felt that they were always blamed for having obesity.

> *I had a very hard time losing weight on my own, I tried a lot of different things, and it was hard, I worked out, ate GI, did weight watchers, everything, and only lost a few pounds, and then when I stopped it, I went up the double. (W1)*

> *It was mostly disappointment and anger at myself like how I could have done this to myself, how could I do this to my body. (W4)*

The women described joint, back and foot pain as well as constant exhaustion from carrying the extra weight that kept them from exercising, hindered them from taking part in employment and isolated them from the outside world. Their health problems became evident when they developed co-morbidities to obesity and these consequences made it clear to them that having obesity was a threat to their health and that they needed to seek help for a more effective weight loss.

> *It felt terrible, so I was sad, really, I felt bad like…. you realized that this is not good either and I was not old, I was 25 years old and could not run up a hill. (W3)*

The women feared that adverse effects on health from having obesity would threaten their abilities to parent by shortening their life span and diminishing capability to be physically active with their children.

> *Now my excess weight will eventually affect my heart and I have a child who was 4 at the time. Who will take care of her if I have a heart attack or stroke and die and disappear? I want to see her grow up and that's what made me do the surgery, that was the last straw. (W2)*

For peer review only

But it was also that the children said, "but mother why are you so tired, why don't you want to do anything, and you are boring, and you never want to do anything", and you heard it all the time. (W4)

Several of the women described difficulties in becoming pregnant before surgery and that they were aware that having obesity could lead to subfertility and reproduction-related difficulties. They feared that having obesity could hinder them from being able to have children of their own.

We tried for several years actively (to conceive) and it did not work, by itself, so we had to seek help in the end. However, I knew that the first thing they will say is that you both need to lose weight. (W5)

Dealing with other people's opinions and society norms

The women expressed how dealing with other people's opinions of them and their weight affected the way that they felt about themselves. They did not want to be around other people and they felt that the stigma associated with being overweight made them less valued. The lack of knowledge concerning the causes of obesity among the public led the women to feel shame for their situation, weight, and appearance.

I thought that nothing was fun when I was big, I didn't want to go out, I didn't want to meet people, I kept to myself a lot, because I felt that no, I had no business being around others, I didn't feel nice enough to be around other people. (W2)

I have knowledge about my operation, about me, so it's probably very difficult for others who haven't had to be in this, in what it is to have obesity, and how it is to have surgery, and the knowledge is very low among others…including healthcare personnel. (W8)

For peer review only

Many of the women presented their experiences of being singled out for having obesity. The women felt shame and worthlessness from their body size and evaded unwanted attention from others because they felt that the norms of society determined a person's value based on how attractive they are perceived to be in a social group.

> *It was mostly that I had the feeling that I was being watched when I was out among people, and whether I was in a galleria or in town, just going out and having a coffee with a friend, I felt that people were staring at me because I was so big, uh, and therefore, I withdrew to stay away from being among people. (W4)*

> *Being fat is ugly, you've been taught that since you were little by society, that you're outside the norm, and that's probably what's ingrained in your mind, that you're a little less worthy, so to speak. (W5)*

Social media's influence on stigma was expressed repeatedly by the interviewed women. The harsh and mean-spirited comments from anonymous persons could have serious effects on the women's self-esteem and mental health and led to them feeling even more isolated from society.

> *Post a picture on social media, and people comment on whether you looked good or not...I think there was a lot of cyberbullying, like back then, if you didn't look good you were told so.... a lot of that started then...it probably started in my generation. (W7)*

The women feared that media's influence on body image could affect the way their children perceived themselves or were treated and they expressed a need for more knowledge concerning obesity and bariatric surgery in order to reduce stereotypes.

For peer review only

I think that it's hard to change with people, actually, I can see as well, um, one of my children, who is not at all overweight, at all, has expressed now quite recently that she's not comfortable with her body, and that she feels fat. (W8)

Life has changed in a positive way

The women that were interviewed expressed that surgery led to their lives being changed in a positive way. The decreased weight burden led to increased energy and improved mobility with greater endurance for physical activity which allowed for more healthy lifestyles as well as feeling more motivated to be socially active.

I think I'm happier as a person, more grateful, I'm trying to, like, take advantage of life, I'm going to, like, take care of myself more with both diet and exercise, I'm grateful that I got this chance. (W1)

Improved endurance and mobility with reduced musculoskeletal pain allowed the women to be employable again and improved their economic situation, including potential for career advancement. Often, there had been several failed attempts previously to rehabilitate to work due to exhaustion and physical pain.

It made a big difference at the workplace because I had back pain. That is the most important thing about having the operation, is that I can live a life again. (W9)

Family life and relationships with their children were enriched after the weight loss from surgery. The women talked about how increased energy and mobility made it possible for them to be the parents they envisioned by playing with their children and taking part in games, outdoor activities and visits to amusement parks without fear of difficulties from their body size.

For peer review only

Everything has become so much easier since I got rid of the extra kilos that were there and were bothering me before. Everything has become a lot easier, yes, I was able to socialize and activate my child in a completely different way too. (W2)

The women explained the importance of clothing and how being able to fit into the current trendy styles included them into society as well as the ability to share clothing with others bringing feelings of fellowship and solidarity. The reduced clothing size after surgery was a reminder of their successful weight loss and a motivator to continue their healthy lifestyles.

It is so clear that it is a boost, that you can choose more clothing and any clothing store, that you are part of society.

You don't feel like the elephant in the room anymore, you can go to a normal store and buy clothes, so it's changed a lot. (W7)

The women described how male-pattern fat dispersion dissipated after surgery and that they experienced more distinct female contours with a defined waist and hips. They felt more confident having more feminine characteristics and enjoyed that clothing fit their figures in a more complementary way. Restoration of menstrual cycles and decreased intensity of menstrual symptoms, such as pain and bleeding, was experienced by a few participants.

At my heaviest my body shape very square, and no waist and not very much shape at all, hips and shoulders, everything was about the same width, and that's something that I noticed…. that I had more defined body parts, so it's a change, purely in terms of appearance. (W6)

I think it was six months, seven months and then the period came back, and it was really a sign that the system is working, so, so that, after a year had passed,

For peer review only

at an annual checkup we were told that now it was ok like, that now we could try to get pregnant (W10)

I used to have quite a heavy period, had a lot of period pain, and things like that, and it has decreased and gotten better, I don't have as much pain, everything has become a little easier (W7)

Sex lives were improved after weight loss and the women explained how the increase in energy, reduced body size and enhanced self-confidence increased their sex drive and motivation for taking part in sexual activities. The women described that they no longer found it uncomfortable to show their bodies without clothing.

I get turned on to things more easily, it's not the same, before it was like I went back and thought no... I don't want to...so there have been some changes now, that the body reacts differently than what it did before...and it has, it's nice in a way, because before I thought there was something wrong with me. (W7)

Accepting inner self and bodily changes

The women experienced many physical alterations after surgery and felt it was difficult to understand, adjust to and accept everything that occurred which led to a feeling of their mind not keeping up with the physical changes in their bodies. They felt that they were still the same size as before their surgery and were often only reminded when they purchased clothing and had to buy much smaller sizes.

The brain doesn't react with what's happening, you don't stand in front of the mirror all the time and keep track, at least not me, the brain just doesn't keep up, that everything changes. (W9)

For peer review only

The head did not keep up with the body, so it takes longer than the body to keep up with the journey. (W4)

It was important for the interviewed women to maintain the same personality and way of thinking as before the surgery with the importance of not allowing weight loss and a changed exterior to influence their opinions of themselves. The women reacted strongly to the more positive treatment they experienced based on their looks since they felt they were the same person on the inside.

I have the attitude that weight has nothing to do with who I am, it has nothing to do with me, I'm still the same person whether I weigh so much or so little. (W10)

There were side effects of surgery that the women dealt with in different ways. The resulting excess skin from decreased fat tissue reminded the women of their previous weight, contributed to a continued large body size, and led to certain hinders in finding clothing that fit properly and at times difficulty with hygiene. The women talked about a tolerance for dumping symptoms and early satiety and accepted that it was part of the effectiveness of the surgery.

You look like you weigh more than you do because you have so much excess skin. (W1)

I get heart palpitations sometimes, so you are physically reminded that you have been careless with food, so it is a huge change that the body does not react to food in the same way at all as before the operation. Now it reacts when you eat too much, or you can feel a little like maybe you shouldn't have eaten that type of food. (W8)

For peer review only

DISCUSSION

This study was designed to explore the lived experiences of women with obesity before and after undergoing bariatric surgery and possible effects of changed sex hormone levels. The principal findings reflected the recognition of an unhealthy body weight as a motivation for surgery and that dealing with society's norms and other people's opinions had a profound effect on their quality of life and mental health before surgery. The women also described the changes after surgery that had a positive influence on their daily lives as well as challenges in adapting mentally to the physical alterations after weight loss.

Although hormonal changes were not a central focus in the women's stories, they mentioned related physical alterations that could be interpreted as hormonal effects that became a part of the whole experience. For example, effects related to sex hormones such as difficulty conceiving prior to surgery as well as reduction of male pattern fat dispersion and improved sexual function after surgery and weight loss were mentioned. However, despite repeated attempts to return a focus to hormones and postoperative changes, the women emphasized preoperative challenges involving living with obesity as well as post-operative benefits concerning improved physical function, quality of life as well as adapting to changes appearances.

Prior to surgery, adverse social experiences based on body size led to isolation and avoidance of social contacts potentially increasing the risk of developing mental health issues, such as depression and anxiety [27]. Stigmatizing attitudes in society have been found in previous studies to have a basis in a lack of knowledge about the pathogenesis of obesity, as well as believing that obesity is associated with negative stereotypes such as laziness, lower intelligence, lack of motivation and self-control [28]. The existence of stigma is even present within the healthcare field and can lead to the women receiving substandard care [29].

For peer review only

Clothing size symbolized success and being able to wear and share trendy and standard sized garments made women feel included in a larger context. Work apparel was another aspect representing inability to “fit in”. A previous study suggested that smaller clothing sizes signified a healthy body weight and associated with more positive self-esteem and improved body image [30]. Clothing size has also been used in previous studies as a measure of risk for developing comorbidities [31, 32]. The women stated that before surgery, their larger attire was a reminder of their unhealthy weight and that reaching the desired weight and clothing size symbolized their journey from having obesity to obtaining a healthy body.

The rapid weight loss and development of excess skin after surgery led to the “mind not following the body” corroborating previous research that reported difficulties adjusting to the new body appearance and resulting in a discordance in their identity perception [33]. Interestingly, research has shown that patients who postoperatively identified themselves as non-obese were more satisfied with their body size than those patients who had lost weight but still identified themselves as having obesity. Fortunately, women became more satisfied with their body size over time [34]. In a study by Gilmartin women who had undergone bariatric surgery emphasized difficulties in changes in body image and a need for support [35]. A meta-analysis showed difficulties in finding standard follow-up measures that take into account all factors that influence a patient’s satisfaction with their body after surgery [36]. Preoperative information about expected changes and experiences of others, and support postoperatively may enable women to cope with the “mind not following the body”.

Quantitative studies have shown restoration of sex hormone regulation in women after bariatric surgery [37] and experiences likely secondary to that were expressed by several women in this study. Satisfaction with having a defined waist, reduction of central fat dispersion as well as restitution of menstrual cycles, more profound cyclic variations in mood after surgery and better sex life were expressed. This was associated with a concrete feeling of

For peer review only

success with surgery and a reinforcement of the health effects of weight loss on the body's function. A limitation may be that a lack of awareness about the relationship between sex hormones and experienced symptoms may, however, have led to not catching certain aspects of hormonal changes during the interviews leading to a primary emphasis on overall benefits and physical changes after surgery. Another limitation might be the language requirement and exclusion of non-Swedish speaking women who may have enriched the detail and increased transferability of the findings.

In conclusion, this study of women´s lived experiences before and after bariatric surgery provided information suggesting that issues related to changes in body size and other people´s attitudes are dominating. Experiences that could be related to changes in sex hormones were mentioned but not central in the women´s narratives. Findings from this study illustrate the preoperative experiences of women with obesity as well as important changes after surgery and weight loss that may require specific support. Guidance concerning preoperative patient expectations and alterations occurring postoperatively can preferably be included in the information to women seeking bariatric surgery and implemented as a routine during follow-up.

Acknowledgments

We look like to extend our gratitude to the bariatric clinic in Falun for assisting in the recruitment of the study participants.

Author contributions

R.P conceived the idea of conducting the study with support and supervision from C.B., T.O., J.F. and E.A. The theory, methodology and interview guide were discussed by all authors and determined with expert supervision from C.B. R.P. performed the interviews and the transcription and discussed and processed the data under supervision from C.B. Analysis was

For peer review only

carried out by R.P. and C.B. R.P drafted the manuscript which all authors revised into the final manuscript.

Funding

This work was supported by grant number 06000984 from the County Council of Östergötland and grant number 974932 from the County Council of Dalarna, Sweden.

Competing Interests

TO participated in advisory boards and educational activities for Johnson&Johnson and Novo Nordisk unrelated to the submitted article, and reimbursements were directed to his academic institution. All other authors have no competing interests to declare.

Data sharing information

Anonymised data will be made available upon request made to the corresponding author.

Ethics Approval Statement

The study was approved in April 2022 by the Swedish Ethical Review Authority, case number 2022-01708-01. All procedures were in accordance with the ethical standards of the 1964 Helsinki declaration and its later amendments. Written and verbal informed consent was obtained from all study participants. Close consideration was taken to protect identities due to the sensitive subject and the limited number of informants.

Figure 1. Visualize the fusions and their relationship.

For peer review only

REFERENCES

1. World Health Organization. Obesity and overweight. 2017.
2. Guh DP, Zhang W, Bansback N, Amarsi Z, Birmingham CL, Anis AH. The incidence of co-morbidities related to obesity and overweight: a systematic review and meta-analysis. BMC public health. 2009;9:88.
3. Balen AH, Anderson RA. Impact of obesity on female reproductive health: British Fertility Society, Policy and Practice Guidelines. Human fertility (Cambridge, England). 2007;10(4):195-206.
4. Brewer CJ, Balen AH. The adverse effects of obesity on conception and implantation. Reproduction (Cambridge, England). 2010;140(3):347-64.
5. Wei S, Schmidt MD, Dwyer T, Norman RJ, Venn AJ. Obesity and menstrual irregularity: associations with SHBG, testosterone, and insulin. Obesity (Silver Spring, Md). 2009;17(5):1070-6.
6. Broughton DE, Moley KH. Obesity and female infertility: potential mediators of obesity's impact. Fertility and sterility. 2017;107(4):840-7.
7. Catalano PM, Shankar K. Obesity and pregnancy: mechanisms of short term and long term adverse consequences for mother and child. BMJ (Clinical research ed). 2017;356:j1.
8. Silvestris E, de Pergola G, Rosania R, Loverro G. Obesity as disruptor of the female fertility. Reproductive biology and endocrinology : RB&E. 2018;16(1):22.
9. Chu DT, Minh Nguyet NT, Nga VT, Thai Lien NV, Vo DD, Lien N, et al. An update on obesity: Mental consequences and psychological interventions. Diabetes & metabolic syndrome. 2019;13(1):155-60.
10. Adams TD, Davidson LE, Litwin SE, Kim J, Kolotkin RL, Nanjee MN, et al. Weight and Metabolic Outcomes 12 Years after Gastric Bypass. The New England journal of medicine. 2017;377(12):1143-55.
11. Sarwer DB, Spitzer JC, Wadden TA, Mitchell JE, Lancaster K, Courcoulas A, et al. Changes in sexual functioning and sex hormone levels in women following bariatric surgery. JAMA surgery. 2014;149(1):26-33.
12. Paul R, Andersson E, Wirén M, Frisk J. Health-Related Quality of Life, Sexuality and Hormone Status after Laparoscopic Roux-En-Y Gastric Bypass in Women. Obesity surgery. 2020;30(2):493-500.
13. Li YJ, Han Y, He B. Effects of bariatric surgery on obese polycystic ovary syndrome: a systematic review and meta-analysis. Surgery for obesity and related diseases : official journal of the American Society for Bariatric Surgery. 2019;15(6):942-50.
14. Skubleny D, Switzer NJ, Gill RS, Dykstra M, Shi X, Sagle MA, et al. The Impact of Bariatric Surgery on Polycystic Ovary Syndrome: a Systematic Review and Meta-analysis. Obesity surgery. 2016;26(1):169-76.
15. Kolotkin RL, Andersen JR. A systematic review of reviews: exploring the relationship between obesity, weight loss and health-related quality of life. Clinical obesity. 2017;7(5):273-89.
16. Jumbe S, Bartlett C, Jumbe SL, Meyrick J. The effectiveness of bariatric surgery on long term psychosocial quality of life - A systematic review. Obesity research & clinical practice. 2016;10(3):225-42.
17. Dawes AJ, Maggard-Gibbons M, Maher AR, Booth MJ, Miake-Lye I, Beroes JM, et al. Mental Health Conditions Among Patients Seeking and Undergoing Bariatric Surgery: A Meta-analysis. Jama. 2016;315(2):150-63.
18. Silva DA, Coutinho E, Ferriani LO, Viana MC. Depression subtypes and obesity in adults: A systematic review and meta-analysis. Obesity reviews : an official journal of the International Association for the Study of Obesity. 2020;21(3):e12966.

For peer review only

19. Ogden K, Barr J, Rossetto G, Mercer J. A "messy ball of wool": a qualitative study of the dimensions of the lived experience of obesity. BMC psychology. 2020;8(1):67.
20. Haga BM, Furnes B, Dysvik E, Ueland V. Putting life on hold: lived experiences of people with obesity. Scandinavian journal of caring sciences. 2020;34(2):514-23.
21. Coulman KD, MacKichan F. Patients' experiences of life after bariatric surgery and follow-up care: a qualitative study. 2020;10(2):e035013.
22. Lier H, Aastrom S, Rørtveit K. Patients' daily life experiences five years after gastric bypass surgery--a qualitative study. Journal of clinical nursing. 2016;25(3-4):322-31.
23. Gadamer H-G. Truth and method: A&C Black; 2013.
24. Kvale S, Brinkmann S. InterViews : learning the craft of qualitative research interviewing. Los Angeles: Sage Publications; 2009.
25. Patton MQ. Qualitative Research & Evaluation Methods: integrating theory and practice. Thousand Oaks, CA.: SAGE Publications, Inc.; 2015.
26. Paul R, Drott J, Olbers T, Frisk J, Andersson E. Motherhood and motivations for bariatric surgery - a qualitative study. Human fertility (Cambridge, England). 2022:1-9.
27. Sarwer DB, Fabricatore AN, Eisenberg MH, Sywulak LA, Wadden TA. Self-reported stigmatization among candidates for bariatric surgery. Obesity (Silver Spring, Md). 2008;16 Suppl 2:S75-9.
28. Friedman KE, Ashmore JA, Applegate KL. Recent experiences of weight-based stigmatization in a weight loss surgery population: psychological and behavioral correlates. Obesity (Silver Spring, Md). 2008;16 Suppl 2:S69-74.
29. Phelan SM, Burgess DJ, Yeazel MW, Hellerstedt WL, Griffin JM, van Ryn M. Impact of weight bias and stigma on quality of care and outcomes for patients with obesity. Obesity reviews : an official journal of the International Association for the Study of Obesity. 2015;16(4):319-26.
30. Petroff AB, Martz DM, Webb RM, Galloway AT. Predicting ideal body mass index: what does clothing size have to do with it? Body image. 2011;8(2):126-34.
31. Hughes LA, Schouten LJ, Goldbohm RA, van den Brandt PA, Weijenberg MP. Self-reported clothing size as a proxy measure for body size. Epidemiology (Cambridge, Mass). 2009;20(5):673-6.
32. Han TS, Gates E, Truscott E, Lean ME. Clothing size as an indicator of adiposity, ischaemic heart disease and cardiovascular risks. Journal of human nutrition and dietetics : the official journal of the British Dietetic Association. 2005;18(6):423-30.
33. Souza ALL, de Souza PM, Mota BEF, Xavier CLF, Santiago FG, Oliveira JS, et al. Ghost Fat: Altered Female Body Perception After Bariatric Surgery. Perceptual and motor skills. 2022:315125221133868.
34. Perdue TO, Schreier A, Swanson M, Neil J, Carels R. Evolving self view and body image concerns in female postoperative bariatric surgery patients. Journal of clinical nursing. 2018;27(21-22):4018-27.
35. Gilmartin J. Body image concerns amongst massive weight loss patients. Journal of clinical nursing. 2013;22(9-10):1299-309.
36. Ivezaj V, Grilo CM. The complexity of body image following bariatric surgery: a systematic review of the literature. Obesity reviews : an official journal of the International Association for the Study of Obesity. 2018;19(8):1116-40.
37. Sarwer DB, Wadden TA, Spitzer JC, Mitchell JE, Lancaster K, Courcoulas A, et al. 4-Year Changes in Sex Hormones, Sexual Functioning, and Psychosocial Status in Women Who Underwent Bariatric Surgery. Obesity surgery. 2018;28(4):892-9.

For peer review only

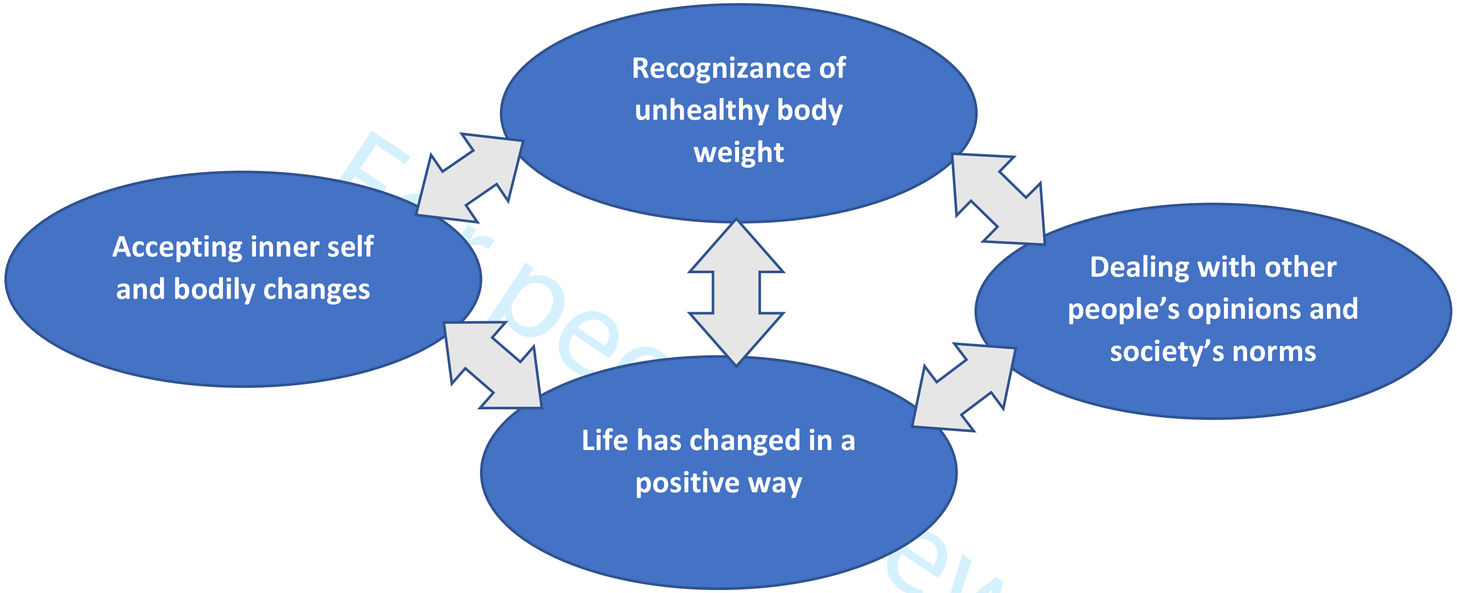

For peer review only

COREQ (COnsolidated criteria for REporting Qualitative research) Checklist

A checklist of items that should be included in reports of qualitative research. You must report the page number in your manuscript where you consider each of the items listed in this checklist. If you have not included this information, either revise your manuscript accordingly before submitting or note N/A.

Topic	Item No.	Guide Questions/Description	Reported on Page No.
**Domain 1: Research team and reflexivity**			
Personal characteristics			
Interviewer/facilitator	1	Which author/s conducted the interview or focus group?	
Credentials	2	What were the researcher's credentials? E.g. PhD, MD	
Occupation	3	What was their occupation at the time of the study?	
Gender	4	Was the researcher male or female?	
Experience and training	5	What experience or training did the researcher have?	
Relationship with participants			
Relationship established	6	Was a relationship established prior to study commencement?	
Participant knowledge of the interviewer	7	What did the participants know about the researcher? e.g. personal goals, reasons for doing the research	
Interviewer characteristics	8	What characteristics were reported about the inter viewer/facilitator? e.g. Bias, assumptions, reasons and interests in the research topic	
**Domain 2: Study design**			
Theoretical framework			
Methodological orientation and Theory	9	What methodological orientation was stated to underpin the study? e.g. grounded theory, discourse analysis, ethnography, phenomenology, content analysis	
Participant selection			
Sampling	10	How were participants selected? e.g. purposive, convenience, consecutive, snowball	
Method of approach	11	How were participants approached? e.g. face-to-face, telephone, mail, email	
Sample size	12	How many participants were in the study?	
Non-participation	13	How many people refused to participate or dropped out? Reasons?	
Setting			
Setting of data collection	14	Where was the data collected? e.g. home, clinic, workplace	
Presence of non-participants	15	Was anyone else present besides the participants and researchers?	
Description of sample	16	What are the important characteristics of the sample? e.g. demographic data, date	
Data collection			
Interview guide	17	Were questions, prompts, guides provided by the authors? Was it pilot tested?	
Repeat interviews	18	Were repeat inter views carried out? If yes, how many?	
Audio/visual recording	19	Did the research use audio or visual recording to collect the data?	
Field notes	20	Were field notes made during and/or after the inter view or focus group?	
Duration	21	What was the duration of the inter views or focus group?	
Data saturation	22	Was data saturation discussed?	
Transcripts returned	23	Were transcripts returned to participants for comment and/or	

For peer review only

Topic	Item No.	Guide Questions/Description	Reported on Page No.
		correction?	
**Domain 3: analysis and findings**			
Data analysis			
Number of data coders	24	How many data coders coded the data?	
Description of the coding tree	25	Did authors provide a description of the coding tree?	
Derivation of themes	26	Were themes identified in advance or derived from the data?	
Software	27	What software, if applicable, was used to manage the data?	
Participant checking	28	Did participants provide feedback on the findings?	
Reporting			
Quotations presented	29	Were participant quotations presented to illustrate the themes/findings? Was each quotation identified? e.g. participant number	
Data and findings consistent	30	Was there consistency between the data presented and the findings?	
Clarity of major themes	31	Were major themes clearly presented in the findings?	
Clarity of minor themes	32	Is there a description of diverse cases or discussion of minor themes?	

Developed from: Tong A, Sainsbury P, Craig J. Consolidated criteria for reporting qualitative research (COREQ): a 32-item checklist for interviews and focus groups. *International Journal for Quality in Health Care*. 2007. Volume 19, Number 6: pp. 349 – 357

Once you have completed this checklist, please save a copy and upload it as part of your submission. DO NOT include this checklist as part of the main manuscript document. It must be uploaded as a separate file.

For peer review only
